# Gender Determination Using Index and Ring Finger Linear Measurements in North Indian Population: A Cross-Sectional Study

**DOI:** 10.7759/cureus.54459

**Published:** 2024-02-19

**Authors:** Prabhpreet Kaur, Pratham Mittal, Harleen Kaur, Jyoti Kiran, Simarjeev Singh, Reeturaj Medhi

**Affiliations:** 1 Department of Oral and Maxillofacial Pathology, Genesis Institute of Dental Sciences and Research, Firozpur, IND; 2 Department of Oral and Maxillofacial Surgery, Dantya Clinic Dental & Cosmetic, Chandigarh, IND; 3 Department of Oral Medicine and Radiology, Gian Sagar Dental College and Hospital, Patiala, IND; 4 Department of Oral and Maxillofacial Pathology, Vyas Dental College and Hospital, Jodhpur, IND

**Keywords:** gender determination, sex, ring finger, index finger, forensic anthropology

## Abstract

Background: In the fields of medico-legal matters and bio-archaeological settings, gender evaluation plays a pivotal role in the initial stages of human identification. Approximately half of the population at risk is excluded when gender is determined, making it the most essential factor for identification. When it comes to medico-legal matters and bio-archaeological settings, gender evaluation is a crucial initial step in human identification. Traditional gender determination procedures, such as skull and pelvic analysis, may be hindered by fragmentary human remains that have been degraded by various forms of inhumation or physical assaults.

Aim: To investigate sexual dimorphism, this study examined the ratio of index finger length to ring finger length.

Materials and method: The lengths of the index and ring fingers were measured and the ratios between them were calculated for both hands separately. Applications of IBM SPSS Statistics for Windows, Version 16.0 (Released 2007; IBM Corp., Armonk, New York, United States) included Student's t-test and Levene's test.

Results: According to the study, women's index finger-to-ring finger length ratios were much longer than men's. The ratio of index finger to ring finger length was significantly different between the sexes on both sides of the hand (p<0.001). In terms of the right hand, the threshold value was 0.9666 for men and 0.9952 for females, while in terms of the left hand, the values were 0.9638 and 0.9920, respectively.

Conclusion: With an advancing front in this arena on gender determination, the use of digits has become an additional source of support to physical anthropologists for bio-archaeological surveys and to forensic experts for use in medico-legal investigations for fragmentary remains received during investigatory trials.

## Introduction

The field of forensic anthropology deals with the identification of mixed-up or misidentified bones in a legal context. Learning a person's ancestry, sex, age, and height is the first step in the identification process [1,2]. As a field, forensic anthropology has long struggled with the difficult problem of distinguishing between mixed and altered remains. Tragedies involving large numbers of people and murders where the victims' bodies are purposefully dismembered to prevent identification, both require the identification of such remains. The determination of a deceased individual's identity holds substantial significance for investigators in cases where bodies have undergone extensive mutilation, as it aids in the establishment of a criminal offense [3].

The field of forensic anthropology is primarily focused on the identification of human remains after death, specifically within a medico-legal framework [4,5]. The investigation of a single hand may help determine important details like age, sex, height, and race via the use of somatometry, osteology, and radiology. The determination of sex is one of the most critical aspects of defining an individual's identity, as it eliminates almost half of the population from the pool of potential candidates [6,7].

There is a lack of extensive study on the use of hand measurements for sex determination, although researchers have attempted to do so using tiny hand bones. Disregarding factors like age, height, and weight, sexual dimorphism may be ascertained by measuring the measurements of the fingers and feet, as well as the interdigital ratios. A key sign for diagnosing sexual dimorphism is the use of anthropometric measurements of hand and foot proportions, particularly length and width [8-10].

Numerous empirical studies have provided compelling evidence linking the ratio of index and ring finger lengths to various physiological and behavioral traits [11]. The objective of this study is to statistically employ parameters like the length of hand fingers, the hand bone length, and the ratio between finger lengths to determine the gender of individuals.

## Materials and methods

After receiving approval from the ethics committee, the study was carried out at the Oral and Maxillofacial Pathology and Forensic Odontology Departments of Genesis Institute of Dental Sciences and Research, Ferozepur, Punjab, India. The research comprised 400 participants, 200 of whom were male and 200 of whom were female, and their ages ranged from 18 to 70. The majority of the subjects were North Indians, hailing from states like Jammu and Kashmir, Punjab, Haryana, Himachal Pradesh, and Delhi.

Inclusion criteria

The research comprised healthy individuals who had not had any surgical operations involving the index or ring finger of either hand, as well as any catastrophic traumas, such as amputations or developmental abnormalities.

Index and ring finger length measurements

**Figure 1:**
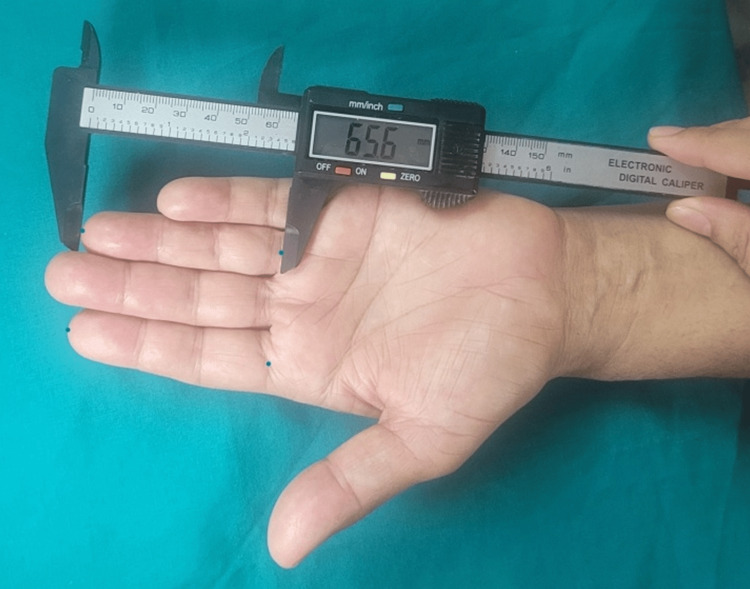
Measurements using a digital caliper

The participant was made to sit comfortably with their hands placed on a flat surface for finger length measurement. Those wearing rings and other ornaments on their fingers were instructed to remove them to see the crease of their fingers for accurate readings. The hands were laid flat on the table, palms up, fingers touching closely. A fissure was found in one of the fingers. There are two folds on a ring finger. For measuring, the second crease near the palm was used. Figure 1 shows the results of the measurements taken using a digital caliper.

A caliper ruler was used with teeth positioned at the midpoint of the proximal-most crease at the base of one finger and the most forwardly situated point (tip) in the midline of the other finger. In both instances, the same investigator recorded the measurements to the closest millimeter, and there were no discernible discrepancies between the results. The other hand went through the same process [9].

The ratio of the index finger to the ring finger was determined to the fourth digit of a decimal for both hands, respectively, using the following formula: index finger length/ring finger length.

Statistical analysis

The data that was collected was examined with the help of IBM SPSS Statistics for Windows, Version 16.0 (Released 2007; IBM Corp., Armonk, New York, United States). We used the Student's t-test to look for differences between the sexes and to compare the ratio of each participant's two hands. A sectioning point was defined as the average of the sexes' ring finger ratios and index fingers to determine the sample's sex. Sectioning point analysis and trial and error led to the determination of a dividing line (cut-off point) for the index and ring finger ratio between the sexes. The sectioning point is equal to the mean male value + the mean female value / 2.

## Results

The average age of the 400 respondents analyzed in the research was 30.87 years for males and 24.19 years for females (Table 1).

**Table 1:** Age of subjects based on gender

Gender	Mean	N	Standard deviation	Minimum	Maximum	Standard error of the mean
Male	30.8650	200	7.97400	18.00	61.00	.56385
Female	24.1850	200	6.84581	20.00	58.00	.48407
Total	27.5250	400	8.14064	18.00	61.00	.40703

Table 2 displays the descriptive data for both boys and females concerning index finger length (IFL), ring finger length (RFL), and the ratio of IFL to RFL (IFL/RFL).

**Table 2:** Index finger and ring finger lengths of right and left hands, and associated index finger and ring finger ratios of both hands. RFL: Ring finger length; IFL: Index finger length

Variable	Gender	N	Mean	Standard deviation	Standard error of the mean
Index finger length (right hand)	Male	200	72.2550	5.28580	.37376
	Female	200	67.7700	3.99586	.28255
Ring finger length (right hand)	Male	200	74.7550	5.01806	.35483
	Female	200	68.1850	4.35033	.30762
Index finger length (left hand)	Male	200	72.2950	5.21960	.36908
	Female	200	67.6600	4.03806	.28553
Ring finger length (left hand)	Male	200	75.0050	5.05199	.35723
	Female	200	68.2750	4.08446	.28881
Right hand (RFL-IFL)	Male	200	2.5000	2.31881	.16396
	Female	200	.4150	2.74416	.19404
Left hand (RFL-IFL)	Male	200	2.7100	2.30488	.16298
	Female	200	.6150	2.70060	.19096
Right-hand ratio	Male	200	.9666	.03106	.00220
	Female	200	.9952	.04024	.00285
Left-hand ratio	Male	200	.9638	.03069	.00217
	Female	200	.9920	.03962	.00280

When looking at men specifically, researchers discovered that RFL was higher than IFL on average. When looking at women, the results were quite similar. The research showed that compared to men, women had a longer ratio of the index finger to ring finger length.

The research discovered a noteworthy disparity in the ratio of the lengths of the index finger and ring finger between the sexes for both the index and ring fingers (p<0.001 ). Furthermore, the ratio of the length of the index finger to that of the ring finger varied significantly (p<0.001 ) between the right and left hands of females (Table 3).

**Table 3:** Levene's test results of the variables.

Variable		Levene's test for equality of variances		t-test for equality of means						
		F	p-value	t	Degrees of freedom (Df)	p-value	Mean difference	Standard error of the difference	95% confidence Interval of the difference	
									Lower	Upper
Right-hand IFL	Equal variances assumed	19.774	<0.001	9.572	398	<0.001	4.48500	.46854	3.56387	5.40613
	Equal variances not assumed			9.572	370.454	<0.001	4.48500	.46854	3.56366	5.40634
Right-hand RFL	Equal variances assumed	8.657	0.003	13.990	398	<0.001	6.57000	.46961	5.64678	7.49322
	Equal variances not assumed			13.990	390.152	<0.001	6.57000	.46961	5.64672	7.49328
Left-hand IFL	Equal variances assumed	21.748	<0.001	9.933	398	<0.001	4.63500	.46664	3.71762	5.55238
	Equal variances not assumed			9.933	374.383	<0.001	4.63500	.46664	3.71744	5.55256
Left-hand RFL	Equal variances assumed	14.869	<0.001	14.650	398	<0.001	6.73000	.45938	5.82689	7.63311
	Equal variances not assumed			14.650	381.274	<0.001	6.73000	.45938	5.82677	7.63323
Right-hand RFL-IFL	Equal variances assumed	7.083	0.008	8.207	398	<0.001	2.08500	.25404	1.58557	2.58443
	Equal variances not assumed			8.207	387.221	<0.001	2.08500	.25404	1.58553	2.58447
Left-hand RFL-IFL	Equal variances assumed	10.059	0.002	8.345	398	<0.001	2.09500	.25105	1.60144	2.58856
	Equal variances not assumed			8.345	388.410	<0.001	2.09500	.25105	1.60140	2.58860
Ratio right hand	Equal variances assumed	15.205	<0.001	-7.951	398	<0.001	-.02858	.00359	-.03564	-.02151
	Equal variances not assumed			-7.951	373.983	<0.001	-.02858	.00359	-.03565	-.02151
Ratio left hand	Equal variances assumed	20.697	<0.001	-7.954	398	<0.001	-.02819	.00354	-.03516	-.02122
	Equal variances not assumed			-7.954	374.559	<0.001	-.02819	.00354	-.03516	-.02122

The cut-off values for differentiating between males and females were 0.9666 and 0.9952 for the right hand and 0.9638 and 0.9920 for the left hand, derived from the comparison of the average lengths of the index finger and ring finger of both sexes. We did this so that we could tell the difference between male and female hands. The t-test and Levene's test were used to analyze the relationship between IFL:RFL on the right hand and IFL:RFL on the left hand, as well as the index finger length on the right hand, ring finger length on the right hand, index finger length on the left hand, and IFL:RFL on both sides. Whether we assume equal variances or not, we find a statistically significant relationship between the sexes (p<0.001).

In comparison to females, men had a greater frequency of anticipated value. In the right-hand ratio, 65.2% of the initially grouped instances were properly categorized, whereas in the left-hand ratio, 66.2% were correctly classified. All things considered, the accuracy rate for case classification was 64.8% (Table 4).

**Table 4:** Prediction results for correctly classified cases

Gender	Predicted group membership	
	Male %	Female %
Ratio of the right hand		
Male	70.5	29.5
Female	40.0	60.0
Ratio of the left hand		
Male	77.0	23.0
Female	44.5	55.5
Combined ratio		
Male	74.0	26.0
Female	44.5	55.5

## Discussion

In certain scenarios, human bodies may undergo severe damage, resulting in the recovery of fragmented body parts. This causes difficulties for forensic scientists to establish an identity, posing a substantial challenge when resolving diverse medical-legal matters. To determine a person's identification, forensic anthropologists may use a variety of methods. We set out to examine sexual dimorphism by comparing the lengths of the index and ring fingers. The process of delineating parameters of digit development during the stage of adulthood yields a more comprehensive understanding of the diverse range of individual variations [12-14]. Understanding the variations in digit morphology can contribute to the diagnosis of developmental pathologies and anomalies affecting the skeletal and endocrine systems. The numerical values hold significant implications for an individual's developmental traits throughout both the prenatal and postnatal stages. The morphological variables of digital and metacarpal formulae possess functional significance. Authors often assess digit length using radiological or morphometric methods, utilizing digital calipers [15]. In the present study, it was stated that the finger lengths of males were comparatively longer as compared to females. This followed the findings of Sen et al., Kanchan et al., Lippa et al., and Harris et al. [16-19].

Our study stated that the length of the ring fingers was greater as compared to the length of the index fingers. This complied with both males and females. In their studies [17,18], Kanchan et al. and Lippa found the difference to be statistically significant. The length of the index finger and the length of the ring finger were found to be significantly different in the male population (p<0.001) for both hands. In the past, several studies have attempted to ascertain the typical length of fingers in both sexes. Lolli et al. found that men typically have a second finger that is 73.82 mm long, while females typically have one that is 67.77 mm long [15]. The average length of a male's fourth finger was 75.27 mm, and a female's was 68.31 mm. Aboul-Hagag et al., Kanchan et al., and Ibrahim et al. found that ring fingers are usually longer in men than in females and that index fingers are about the same length in both sexes [17, 20-21]. When comparing the two sexes, the current research found that the average ratio of the lengths of the index and ring fingers was significantly different in females (p<0.001) compared to men. Aboul-Hagag et al., Kanchan et al., Ibrahim et al., and Bailey and Hurl all came to similar conclusions in their research [17,20-22].

Our research showed that both sexes maintained a steady ratio of the lengths of their index and ring fingers on their right and left hands. When looking at the average ratio of the lengths of the index and ring fingers on both hands, Sen et al. found no significant results [16]. According to Barrett and Case's research, 2D:4D digit ratios could show population-specific variances, making them an unreliable way to determine someone's gender [23]. We looked at the average ratio of ring finger length to index finger length for both sexes. On one hand, we found that the ratio of the lengths of the index and ring fingers was 0.9666 and 0.9952, while on the other hand, we found that the ratio was 0.9638 and 0.9920. The purpose of this step was to distinguish between the hands of men and women. This study's findings were consistent with those of Aboul-Hagag et al. and Kanchan et al. [17,20].

One way to measure the proportionality of different digits or fingers is to take their lengths from the middle of the bottom crease, where they join to the hand, and then measure to the tip. This measurement is called the digit ratio [24]. Some researchers have hypothesized that androgens like testosterone may affect the relative lengths of two fingers during fetal development. These fingers are the index finger and the ring finger. As a result, the ratio of the second digit and the fourth digit (2D:4D ratio) has been proposed as a basic measure of the amount of androgens exposed to a developing fetus, with lower ratios suggesting a higher level of exposure [25]. A 2011 study by Zheng and Cohn found that the control of the 2D:4D ratio in mice is impacted by the interaction of androgen and estrogen signaling during a particular period of digit development. The development of human digits during the prenatal period is commonly believed to take place by the 13th week of gestation. The bone-to-bone ratio in the fingers also stays rather constant from this developmental stage until adulthood. During this specific period, the fourth finger grows faster when the growing fetus is exposed to androgens at a concentration thought to represent sexual dimorphism. When looking at the 2D:4D ratio in female-to-male dizygotic twins, this phenomenon becomes clear because the female twin gets exposed to a lot of androgens from her male twin while she's pregnant, which causes the 2D:4D ratio to be much lower [26].

The study's limitations include that it was primarily focused on a specific population group, limiting the generalizability of the findings to individuals from diverse ethnic backgrounds. The sample size, although yielding significant results, could benefit from expansion for enhanced robustness. Additionally, relying solely on finger length ratios for gender determination might be further refined by incorporating additional anatomical measurements or cross-validation through independent studies. Age variations within the sample were not considered, which could influence the accuracy of gender determination in diverse age groups. Even with these flaws, our study adds to the growing field of figuring out gender and can help physical anthropologists in bioarchaeological surveys and forensic experts in medico-legal investigations involving fragmented remains during investigatory trials.

## Conclusions

With the increasing utility of forensic science and anthropology in gender determination to deal with mutilated and fragmented remains while investigating medicolegal cases, the use of digits as an aid to determining the gender of an individual has come up as an additional support to physical anthropologists during bio-archaeological surveys and to forensic experts in medico-legal investigations. More such studies should be undertaken on other population groups to develop a database for further credibility of the results obtained.

As we expand our understanding of skeletal variations among different races and populations, it becomes evident that further studies on various population groups are needed to establish a robust and comprehensive database. By researching on a global scale, we can enhance the credibility and applicability of the results obtained.
